# Structures of Gold(I) and Silver(I) Thiolate Complexes of Medicinal Interest: A Review and Recent Results

**DOI:** 10.1155/MBD.1999.201

**Published:** 1999

**Authors:** Helen E. Howard-Lock

**Affiliations:** 1 Laboratories for Inorganic Medicine McMaster University Hamilton, ON L8S 4M1 Canada

## Abstract

We review the crystal structures, electrospray ionization mass spectra (ESI-MS) data and chiral HPLC data for the racemic and optically pure mononuclear AuL2 complexes, and for racemic [AuLI]n and optically pure [AgLII]n polymers (LI = thiomalate, LII = **D**-penicillamine). We postulate an equilibrium between polymeric, mononuclear and free ligand species for [AuLII]n (gold sodium thiomalate or GST). The ESI-MS results clearly show a tetrameric principal species in the 1:1 gold polymers, [AuLI]n. For the 1:1 silver: **D**-penicillamine complex, [AgLII]n, a non-molecular crystal of double helical structure, the ESI-MS results show multi-ligand-silver species, including tetramers, pentamers and hexamers. Other, relevant gold, silver and copper complexes are compared.


### STRUCTURES OF GOLD(I) AND SILVER(I) THIOLATE COMPLEXES OF MEDICINAL INTEREST: A REVIEW AND RECENT RESULTS

Helen E. Howard-Lock

Laboratories for Inorganic Medicine,
McMaster University, Hamilton, ON, Canada L8S 4M1

**Abstract**: We review the crystal structures, electrospray ionization mass spectra (ESI-MS) data and chiral HPLC data for the racemic and optically pure mononuclear AuL2 complexes, and for racemic [AuLI]n and optically pure [AgLII]n polymers (LI = thiomalate, LII = **D**-penicillamine). We postulate an equilibrium between polymeric, mononuclear and free ligand species for [AuLII]n (gold sodium thiomalate or GST). The ESI-MS results clearly show a tetrameric principal species in the 1:1 gold polymers, [AuLI]n. For the 1:1 silver: **D**-penicillamine complex, [AgLII]n, a non-molecular crystal of double helical structure, the ESI-MS results show multi-ligand-silver species, including tetramers, pentamers and hexamers. Other, relevant gold, silver and copper complexes are compared.

### Aurophilicity

Chemists should stop expressing surprise when short Au···Au distances appear in structures of gold complexes. In a review of about 700 structures containing Au-S bonds, Au-Au contacts range from 2.50 to 4.00 Å. It is difficult to define a Au-Au contact as bonded or non-bonded by distance alone, but gold-gold interactions occur within narrow distance ranges, and the orientation of gold atoms in crystals are characterized by specific angular geometries. Arbitrarily the distances 2.55-3.10 Å have been assigned to Au-Au bonds; 2.90-3.30 Å to Au···Au non-bonded intramolecular interactions; and 2.80-3.40 Å to intermolecular contacts between molecular species with a single gold atom.[1] There is some overlap between categories. For comparison, in the metal the Au-Au bond is 2.89 Å and the nominal van der Waal's distance is 3.40 Å.

Relativistic effects have been discussed in detail by Pyyckko[2] and their inclusion in calculations gives an ionic radius for gold of 62 pm, much less than previous typical literature values, and moreover, less than that of silver. See Table I. The Au···Au bond of ~ 3.00 Å has an energy the order of that of the hydrogen bond, up to 25 - 37.5 kJ $mol^{-1}$ for gold(I) complexes,[3] and therefore one can expect it to determine the packing of a crystal. Aurophilicity may be described as a correlation effect strengthened by relativistic effects. The relativistic contraction of the 6s orbital makes gold unique among unusual elements and in fact some gold bonds are shorter than corresponding silver bonds. Cuprophilicity and argentophilicity are also known, as mentioned below.

Table I. M(I) Ionic Radii and Bond Lengths, pm[4]

| | Cu | Ag | Au |
|---|---|---|---|
| typical literature values, ionic radii | 46 | 67 | 137 |
| Relativistic values, ionic radii | 49 | 68 | 62 |
| metal-hydrogen M-H bond length | 146 | 162 | 152 |
| metal-sulfur S-M-S bond length | 205 | 237 | 228 |

M(I) radii and bond lengths for two-coordinate structures, *LeBlanc, D.J. Ph.D. Thesis, McMaster University, (1996).*

### Polymeric complexes of gold(I) and thiomalic acid

Gold sodium thiomalate (GST) has been marketed in Canada for over 60 years as an anti-rheumatoid arthritis drug under the name Myochrysine™ (myocrisine, generic). The drug has remained very poorly characterized, and the exact nature of this drug was unknown until now. First of all, the drug as administered is a chemical mixture, from which we have identified eight components.[5] It has been accepted that the major, and active component, is a polymer of disodium thiomalato-S-aurate(I). Various polymeric structures, of both cyclic and open chain form, have been proposed, including hexamers and pentamers. (The nomenclature hexamer, pentamer, tetramer is in common use for gold thio-ligand complexes, to describe six, five or four -Au-S- groups joined together. A tetramer in this sense is actually an eight-membered ring of four -Au-S- pairs.) Secondly, the chirality of the ligand greatly confuses the elucidation of the structure. We have discussed this in detail, with diagrams, for a cyclic hexamer.[5] Similar calculations show the number of diastereomeric components for pentamers to be six; that is, 5***R***, 4***R***1***S***, 3***R***2***S***, 2***R***3***S***, 1***R***4***S***, or 5***S*** stereocentres in the ratio 1:5:10:10:5:1. For tetramers the number of components will be five, 4***R***, 3***R***1***S***, 2***R***2***S***, 1***R***3***S***, 4***S*** in the ratio 1:4:6:4:1. Actually, the 3***R***2***S*** and 2***R***3***S*** diastereomers have three arrangements, and the 2***R***2***S*** have two. For each combination the basic $(\text{-Au-S-})_n$ structural unit remains the same, but the outer conformation of the molecule will differ. The existence of these multi-component "optical sludges" is one of the reasons why these compounds have been so difficult to crystallize. We have always obtained amorphous solids; and in commercial practice myochrisine is obtained as an oil.

Table II is a summary of earlier experimental work that has been carried out on GST, both polymer and monomer, by other groups and ourselves. Determination of the structure by methods such as X-ray Absorption Fine Structure (XAFS) and Wide Angle X-ray Scattering/Differential Anomalous Scattering (WAXS/DAS)[6] revealed that the gold atom is bound to two sulfur atoms with Au-S distances of 2.30 Å and an S-Au-S angle of about 180°. Longer range interactions give Au···Au distances of 3.35 and 5.8 Å which have been interpreted as suggesting that the structure is an open chain polymer and the repeat unit is a pentamer. Modelling by us has shown that these structural parameters also fit a cyclic Au-S pentamer. The next nearest Au···Au distances lie in the range 5.0 - 6.7 Å for each combination depending on the torsion angle; for 90° the distance is 5.8 Å. Other methods of examining the compound, such as Mössbauer spectroscopy, have suggested the presence of two or three different types of gold(1) atom present in the compound. This would be inconsistent with a single cyclic structure, but might be evidence for an open chain with differing gold environments at the middle and ends of the chain. Another possible explanation is that two (or more) cyclic and/or open-chain complexes are present. Chemical analyses of samples of Myochrysine have shown variation in the ligand to gold ratio,[7] in particular differing from the 1:1 ligand to gold ratio required by a ring system. Mary Duarte and I prepared the acid analogue of GST, by the direct reaction of thiomalic acid with chloro(isobutylisonitrile)gold(I); chemical analysis for this complex gave excellent agreement between calculated and observed values for C, H, S, and Au based on a 1:1 compound.[8] It seems likely that GST in solution, and probably in the solid, comprise more than one gold-containing species corresponding to different degrees of polymerization. The number and nature of these species present depends on the concentration, pH and nature of the cation ($Na^+$, $NH_4^+$, etc.).

Mary Duarte and I developed a method for synthesizing optically pure ***R*** and ***S*** thiomalate, and from it, optically pure ***R*** and ***S*** GST.[8] We were unable to obtain crystals, but we did measure the density of the amorphous solids for both racemic and optically pure GST. The solid racemic GST separated gradually over 24 hr into two layers having densities 2.731 (upper layer) and 2.910 (lower layer) g $cm^{-3}$, values about 1.5 times greater than those for mononuclear 1:1 gold(I) thiolate complexes. By means of HPLC with a C18 column, the denser component elutes first, the retention times, RT, being 3.027 and 3.243 minutes. See Table II. These facts are consistent with the presence of oligomers, or polynuclear complexes, of two different sizes. Modelling of various polynuclear structures by Russell Bell shows that the smaller compact structure of the cyclic tetramer or cyclic pentamer have densities closest to the observed values. The mononuclear structures have measured densities of only about 2.00 g $cm^{-3}$. The mass spectra evidence for presence of tetrameric species will be discussed in more detail below. Multi Ag:L species have been observed also for related silver complexes.[9]

We carried out spectroscopic measurements (IR, Raman, UV-Vis and CD), and High Performance Liquid Chromatography (HPLC) on various columns, including chiral columns. HPLC separations both of GST synthesized in our laboratory (from racemic thiomalic acid) and of the commercial drug, show two or three very sharp peaks; sharp peaks are also found for GST synthesized from optically pure enantiomers of thiomalic acid[8]. Similarly, capillary zone electrophoresis separations of GST show a single sharp peak, unless extra thiomalate is added, when a broad band appears. This sharpness of the major peaks suggests that it is caused by a cyclic polymeric species. Addition of excess thiomalate to the rapidly exchanging gold ligand system apparently causes ring-opening and exchange processes such that the polymer is broken up into shorter sections: $[AuL]_n + L = Au_nL_{n+1} \ldots Au_4L_5 \ldots Au_2L_3 \ldots$. The limiting species is the mononuclear $AuL_2$ complex with a 2:1 thiomalate to gold(I) ratio. The open-chain complexes of differing lengths will have a broader range of motions than the cyclic species.

We also carried out tests for biological activity for the ***RS***-, ***R***- and ***S***-GST. The tests used were (i) inhibition of collagen-induced platelet aggregation and (ii) inhibition of mixed lymphocyte response. These tests are important because they are relevant to the immune system and they are likely to correlate with the potential anti-rheumatoid activity of the drug. Concentrations were comparable to the plasma gold levels found in rheumatoid patients being treated with GST (myocrisine), ($2.5 \times 10^{-5}$ - $6 \times 10^{-4}$ M in elemental gold). In each case, ***S***-GST had a dramatically greater inhibitory effect than the ***R***-GST. An important observation is that the results of the biological tests for the ***RS***-GST do not fall half way between those for the ***S***- and ***R***-forms, as might be expected for a simple racemic compound. ***RS***-GST is less inhibitory than either ***R***- or ***S***-GST, but more so than commercially available GST. This is because the polymeric structure contains a mixture of several diastereomers having different optical properties and different biological activities, so that the pure ***R*** and pure ***S*** components will be present in much the lowest concentrations.

Table II: Experimental Work on Polymeric and Mononuclear Gold(I) Thiomalate Complexes, [a, *, +]

| | **Polymeric**[*,+] ***RS*** GST ($[AuL]_n$) | | **Mononuclear*** | |
|---|---|---|---|---|
| | | | ***RS*** $AuL_2$ | ***S*** or ***R*** $AuL_2$ |
| Mössbauer[+] | 2 (or 3) gold environments, probably in different species | | | |
| Structure: | WANES/WAX, *Elder et al.*[+,6] | | X-ray Structure, *this work*[*,10] | |
| Au-S, Å | 2.30 | | 2.277, 2.251 | 2.282, 2.268 |
| S-C, Å | | | 1.79, 1.78 | 1.818, 1.834 |
| S-Au-S, ° | ≅ 180 | | 178.8 | 176.03 |
| Au···Au, Å | 3.35, 5.8, 8.1 | | 4.2 | 7.5 |
| Chemical Analyses * | Au:L , 1:1 [7,8] | | Au:L , 1:2 | Au:L , 1:2 |
| 2 component system: | Upper | Lower | | |
| Observed Density*[,8] g $cm^{-3}$ | 2.731 ***RS*** | 2.910 ***RS*** | 1.96 ***RS*** | 2.05 ***R*** |
| HPLC-C18, RT, min | 3.243 | 3.027 | | |
| Theoretical Density * g $cm^{-3}$ | | | | |
| cyclic tetramer | 2.807 | $Au_4L_4$ | | |
| cyclic pentamer | 2.794 | $Au_5L_5$ | | |
| open chain pentamer | 2.781 | $Au_5L_6$ | | |
| open chain tetramer | 2.589 | $Au_4L_5$ | | |
| HPLC [*,b] | Separation of 2 components, One white, one yellow-green | | | |
| Mass spectra: ESI-MS * | Tetramers in +ve and -ve ion modes Variation of cone voltage | | Monomers $Au_2L_3$ (weak) | |
| $^{13}C$ nmr * | Thiomalates bridge gold atoms | | Terminal thiomalate ligands | |
| IR and Raman * ν Au-S $cm^{-1}$ 367 ν C-S $cm^{-1}$ | 367 595 --- | | 367 595 --- | |
| UV-Visible, $\lambda_{max}$ nm* | 214, 260, 340. | | | |
| Biological tests* Platelet, lymphocyte | ***S*** inhibits more strongly than ***R*** | | | |

[a] L = thiomalate; **this work*; *[+]other work;* [b]For Chiral HPLC, See Table III.
RT = retention time in minutes; $\lambda_{max}$ nm, wavelength in nanometers.

Table III is a summary of the chiral column HPLC data for GST or ($[AuL_1]_n$). Any optically pure species (for example, any polynuclear species, including the cyclic tetramer and the open-chain dimer, as well as the $AuL_2$ complex), would give just one peak. If the compound is racemic (or partially mixed), the number of peaks should depend on the size of the oligomer, and the peaks corresponding to the all ***R*** or all ***S*** will be the weakest. The $AuL_2$ complex would have 3 species, ***RR***, ***RS***, and ***SS*** in a 1:2:1 proportion. The ***RS*** ligand alone would have two peaks corresponding to the ***R*** and ***S*** forms, in a 1:1 proportion.

Table III: Chiral HPLC of GST[a,b]

| ***S*** GST | | ***R*** GST | | ***RS*** GST | | M&B GST | |
|---|---|---|---|---|---|---|---|
| **t, min** | **I, %** | **t, min** | **I, %** | **t, min** | **I, %** | **t, min** | **I, %** |
| | | 3.53 | 45 | | | | |
| | | | | 3.65 | 85.3 | 3.64 | 94 (47) |
| 3.79 | 72.8 | | | | | | |
| | | | | | | 4.00 db | (47) |
| | | | | | | 4.41 | 4.6 |
| 4.82 | 25 | | | 4.83 | 10.7 | | |
| | | | | | | 4.91 | 1.5 |
| | | 5.52 | 55 | 5.51 | 4.0 | | |
| 6.29 | 3 | | | 6.3 | wk, br | | |

[a] Experiments conducted with Chiralpak WH, ecd, EW 0.1462 V, pot +0.21 V. Data reproduced three times at 3 working potentials. The peaks start to coalesce at EW = 0.265 V. Initial materials were GST (or myocrisine, i.e., the $[AuL_1]_n$, 1:1 thiomalic acid to gold polymer): Labels: ***S***-, ***R***-, and ***RS***-GST, our synthesis; M&B GST, May & Baker commercial Myochrysine.
[b] Symbols: t , min = time in minutes; I, % = relative intensity in %; db = doublet, wk = weak, br = broad.

Peaks at retention times of 3.53 min ***R***, 3.79 min ***S***, and 3.65 min ***RS*** can be assigned to the gold complexes. For May and Baker GST (M&B GST), and ***RS***-GST (our synthesis), the main peak ≈3.64 min has the average of the retention times of the first peaks for ***R*** and ***S***. If this were the mononuclear $AuL_2$ species, it is hard to explain the absence of ***RR*** and ***SS*** peaks that should have half of the intensity of the ***RS***. More likely these peaks arise from polynuclear species, for which peaks attributable to pure ***R*** and pure ***S*** forms, and possibly some other mixed forms, are too weak to be observed. An alternative explanation is that there is an exchange process going on within the column so that only an average peak is observed. Peaks at retention times 4.82 min ***S***, 5.52 min ***R***, and 4.83, 5.51 min ***RS*** can be assigned to the ligand unambiguously. Thus the major peaks in the chiral column HPLC of solutions could be assigned to a polynuclear species and the ligand alone. The presence of these two species is reasonable considering the equilibria that exist in solution.

**Mononuclear $AuL_2$ complexes of gold(I) with $L_I$ = thiomalic acid and $L_{II}$ = D-penicillamine**

For a long time we had evidence, from $^1$H nmr studies of the interaction of AuI with thiomalic acid, that we had obtained the $Au(L_I)_2$ anion as well as $(AuL_I)_n$, the 1:1 ligand to gold GST polymer. In 1995-96 we made a breakthrough in establishing a structure for one of the components of GST. Zhixian Wang was able to confirm the existence of the $AuL_2$ anion by isolation of the ammonium salt. He crystallized the mononuclear racemic thiomalate complex of gold(I), pentammonium di(thiomalato-S) aurate(I), (Table IV, compound **I**).[10] One of the interesting features of this structure is that although the overall crystal is racemic, individual anions within a given crystal contain only ***RR*** *or* ***SS*** ligands. Daren LeBlanc then crystallized and determined the structure of the analogous $AuL_2$ anion obtained from the ***S*** thiomalate complex of gold, **II**.[10] The ***R*** complex formed an enantiomorphic crystal having the identical structure. A similar method of preparation was used to allow us to isolate the optically pure ***S*** $Au(L_{II})_2$ complex, **IV**, of gold(I) and D-penicillamine.[11]

It should be noted that D-penicillamine itself is a second line or disease-modifying anti-rheumatoid arthritis drug, like Myochrysine. It has a great many medical uses, the better known being its use in heavy metal, particularly copper, removal. Recently it has been shown to inhibit HIV replication. We can speculate that its gold complex may also turn out to be useful as an anti-arthritis or anti-cancer drug. The size and shape of D-penicillamine are very similar to those of thiomalic acid. The two anions also have superficially similar structures in the crystals. Interestingly, a racemic crystal, **III**, could not be formed when ***RS***-penicillamine was used in the preparation. Instead, enantiomorphic crystals containing all ***RR*** or ***SS*** anions were obtained. Table IV summarizes the structural and spectroscopic features of these $AuL_2$ complexes. For all these compounds, the gold atoms are coordinated to two sulfur atoms from two separate ligands and the Au-S bond lengths lie in the normal range for mononuclear complexes of this type. All other bond lengths and angles are normal. In **I** and **II** there are no short Au···Au distances, but in **IV** three molecules, related by a threefold axis, are in relatively close contact, with Au···Au distances of 3.221 Å. The packing is determined by these aurophilic interactions, as well as by hydrogen-bonding networks formed between amine and carboxylate groups on neighbouring ligands. It must be emphasized that short Au···Au distances are not of themselves evidence for polymerization, since these are mononuclear complexes.

### The Equilibria

In light of the establishment of the existence of a stable monomer, and Sadler's[12] postulated cyclic hexamer structure for the polymer, the work of Schröter and Strähle is of interest.[13] With the achiral ligand 2,4,6-tri(isopropyl)thiophenol they were able to isolate and determine the crystal structures of **both** a monomer and a cyclic hexamer. By a slightly different method our group prepared the same complex, having different packing and solvent of crystallization, and confirmed its cyclic hexameric structure.[14]

Table IV. Structural features of the $AuL_2$ mononuclear complexes ($L_I$ = thiomalate; $L_2$ = penicillamine)

| Complex (Ligand, L) | I (*RS*-Thiomalate) | II (*S*-Thiomalate) | IV (*S*-Penicillamine) |
|---|---|---|---|
| IR, Raman: | | | |
| ν Au-S, $cm^{-1}$ | 367 | 367 | 380 |
| ν C-S, $cm^{-1}$ | 595 | 595 | 610, 560 |
| ν S-H, $cm^{-1}$ | --- | --- | --- |
| X-ray crystal structure: | | | |
| Au-S, Å | 2.277, 2.251 | 2.282, 2.268 | 2.29 |
| S-C, Å | 1.79, 1.78 | 1.818, 1.834 | 1.841 |
| S-Au-S, ° | 178.8 | 176.03 | 178.5 |
| Au···Au, Å | 4,2 | 7.5 | 3.221 |

**I** Steric interaction of the ligands prevented close approach of the S-Au-S cores. The crystals were racemic but all anions were only ***RR*** or ***SS***.

**II** The S-Au-S cores were arranged around the $6_2$ axes, H-bonding defined channels; distance across channel 7.5 Å (Au···Au). ***R*** $Au(L_I)_2$ gave identical, enantiomorphic crystal.

**III** The ***RS*** penicillamine complex produced enantiomorphic crystals containing all ***RR*** or ***SS*** anions. (i.e., **IV**).

**IV** The S-Au-S cores were arranged around 3-fold axis, Au···Au 3.22 Å, aurophilic bonding.

We postulated in 1994[15] that the major equilibrium in GST solutions might be between the $AuL_2$ mononuclear complex and a cyclic hexamer, where $L_I$ is thiomalic acid:

$$6[Au(L_I)_2]^{5-} = [(Au\,L_I)_6]^{12-} + 6(L_I)^{3-}$$

Under normal preparation conditions (i.e., a minimal excess of ligand) this equilibrium should lie well to the right and the hexamer would be the major product. Clearly the interconversion would have to go through components of varying degrees of polymerization which would probably be open chains. More recently, however, we have obtained other evidence that suggests that a tetrameric species is part of the major equilibrium.

One should note in passing that the mononuclear complexes are reasonably stable in the solid state and remain as colourless solids for weeks, if stored in the dark. The thiomalate complex in aqueous solution in air slowly turns yellow. Under the conditions used to sterilize GST (30 min at 100°C), the change is rapid. This change can be suppressed completely if the water is degassed thoroughly (boiling followed by cooling in vacuum) before the gold compound is dissolved, and the solution is kept under vacuum or nitrogen during the sterilization process. Thus the formation of the yellow colour depends on oxidation of the ligand; the change indicates that some monomer is converted to the polymer with the formation of free ligand, which in turn forms disulfide. The oxidized species are responsible for some adverse effects of the drug.

### Comparison of the mass spectra of the drug Myochrysine, the polymer ammonium analogue of GST, and the $AuL_2$ mononuclear complex

From our earliest research on gold complexes we have attempted mass spectral studies of GST, but with little success. When an electro-spray ionization mass spectrometer, ideal for looking at ionic compounds, became available, we tried again.[16] First we looked at the crystallographically-characterized mononuclear species: In addition to the peak of $Au(L_I)_2$ itself, some of the less pure samples exhibited a very small amount of an $Au_2L_3$ species (dimer) and its derived 2:2 ion. We then applied this technique to the ammonium analogue of GST. To our surprise we obtained a relatively clean spectrum that showed not the hexamer but a tetramer; there was no evidence of peaks having higher m/z than the 4:4 species. Similar mass spectra were obtained from a sample of the commercial drug suspended in ammonium hydroxide. Although the spectra were not so clean, because of the impurities in the drug, no peaks at m/z values greater than those for the tetramer were seen. We see the 4:4 species in both positive and negative ion spectra, and with variations of cone voltage. The presence of these fragments was insensitive to cone voltage. Collisional activation MS/MS studies of fragments also showed only tetramer fragments; this suggests that the tetramer was present in solution. For the crystalline solid of the $Au(L_I)_2$ monomer, no tetrameric species were observed in the mass spectra. (Likewise for crystalline $Au(L_{II})_2$, only monomeric species were observed in the mass spectrum.) The main species in the mass spectra for all of the various GST preparations and the ammonium analogue are cyclic tetramers. We think that, on the basis of the mass spectrometric evidence, one of the main components in solution, and possibly in the amorphous solid, of the drug, is also tetrameric. Then the interconversion reaction is

$$4[Au(L_I)_2]^{5-} = 2[L_I\, Au\, L_I\, Au\, L_I]^{7-} + 2\,(L_I)^{3-} = [(Au\, L_I)_4]^{8-} + 4\,(L_I)^{3-}$$

and in the drug this equilibrium lies well over to the right. Whether two dimer anions condense directly to the tetramer, or whether the reaction is through the $Au_3L_4$ anion cannot be stated, but there is little evidence of the latter in the mass spectra. Clearly, in water there will be hydrolytic reactions involving the anion but we have ignored these for simplicity.

### Cyclic tetramers in 1:1 gold thiolate complexes

Since that time we have found in the literature two structures that contain 4:4 thiolate:gold(I) cyclic tetramers (ligand = $SC(SiMe_3)_3^-$, $SSi(Bu^tO)_3^-$),[17,18], demonstrating that such a framework occurs in other gold complexes. Both of these had an essentially square planar $Au_4S_4$ core, with Au-S-Au angles slightly greater than 90°. These, however, were non-ionic and could not be subjected to the ESI-MS studies. Laguna *et al.*[19] have reported a novel tetranuclear gold(I) complex which shows that geometries other than planar are possible with constraining ligands (o-carborane derivatives). The four gold atoms form short contacts, 3.131 Å (transannular), and slightly longer ones at 3.568 and 3.751 Å. The central gold atoms show nearly linear coordination and the peripheral gold atoms show distorted tetrahedral geometry (to two sulfur and two phosphorus atoms). The eight-membered ring adopts a chair conformation.

### Cyclic structures of copper and silver complexes

A number of structural studies of 1:1 thiol ligand to metal complexes for copper and silver have been done and provide some insight into the possible gold complex structures.[20] The cyclic tetrameric structure is very important in thiols of both copper and silver, whereas no cyclic hexamers have been reported for these metals. The degree of polymerization depends on the nature of the ligand: Sterically crowded ligands tend to produce small, compact structures, as small as a cyclic trimer for Ag, while less bulky ligands result in larger ring sizes, including cyclic tetramers and double tetramers (two cyclic tetramers weakly linked), and a cyclic dodecamer for the silver complex of cyclohexanethiol.

### Double helices in gold and silver complexes

In 1996 David Green of our group prepared two gold(I) complexes with the ligands mercaptoacetic acid and 2-mercapto-5-methylbenzimidazole, and obtained the mass spectra for both and a crystal structure for the latter. Aurophilic bonding results in an unprecedented double helix chain polymer structure. The two interlinked helices have a tetrameric repeat unit; a distinctive feature of this structure is that the original ligand has been oxidized to form a trisulfide.

In 1998 Russell Bell[21] obtained the crystal structure and mass spectra for the related 1:1 Ag:**D**-penicillamine complex, $[AgL_{II}]_n$. It also forms a double helix of interweaving chains with a Ag-S bond length of 2.37 Å and Ag···Ag distances of 2.95 - 3.03 Å, a good example of argentophilicity. The ESI-MS studies show the presence of multi-silver-ligand species, including tetramers. The presence of the two silver isotopes $^{107}Ag$ (51.8%) and $^{109}Ag$ (48.2%) (that is, nearly 1:1) means, for example, that a tetranuclear silver unit shows as a pentet of lines of close to 1:4:6:4:1 intensity. These are clearly evident, for example, at m/z 1022.8 ($Ag_4L_4$) and 873 ($Ag_4L_3$). Interestingly, there is also strong evidence of pentameric fragments (sextets at m/z 1128.8, $Ag_5L_4$; 981.6, $Ag_5L_3$) and (weaker) hexameric fragments (heptet at m/z 1236.7, $Ag_6L_4$ ). Nomiya *et al.*[9] observed similar pentets in the ESI-MS of a 1:1 thiomalate:silver complex, and they believe the cyclic tetramer to be a stable subunit of the oligomer.

More recently, Robert Bau[22] has made an important contribution to the crystal structural data of GST. Using a hanging drop vapour diffusion technique and cationic (cesium ion) additives, he obtained a crystal structure for racemic GST (as supplied by Aldrich Chemical Co., and not the commercial drug Myochrysine). The GST crystallizes as a mixed sodium/cesium salt in the tetragonal space group $P\,\bar{4}b2$ (No. 117), $a$ = 18.767(2) and $c$ = 4.798(2) Å. The gold-sulfur backbone of the GST polymer exists as two interweaving spirals or helices. There are two independent gold atoms in the unit cell, one having near linear coordination ($S-Au_1-S$ = 178.9°) and the other distorted from linearity ($S-Au_2-S$ = 169.4°). The molecules pack so as to form an equal number of right-handed and left-handed helices in the unit cell. The left-handed helix is formed exclusively with ***S*** ligands, the right-handed with ***R***. The repeating unit of the chain along the c axis in the unit cell is tetrameric. The shortest Au···Au distances are 3.227 Å, interstrand, and 3.485 Å, intrastrand. See Table V. The average Au···Au distance, 3.356 Å, together with other parameters, agree well with Elder's EXAFS/WAXS analysis. We calculate a density of 2.726 g $cm^{-3}$ for the crystal, if the cations are ignored.[23] This density is very close to the observed density for solid non-crystalline racemic GST, our synthesis. In Table V the structural parameters, chemical analyses and densities for the polymers and mononuclear complexes obtained from GST (gold thiomalate, various sources) are compared.

Table V: Structural Data for Polymeric and Mononuclear Gold(I) Thiomalate Complexes

| | **Polymer** | | **Mononuclear** | |
|---|---|---|---|---|
| | ***RS*** $[AuL]_n$ | | ***RS*** $AuL_2$ | ***S*** or ***R*** $AuL_2$ |
| Structure: | WANES/WAX, *Elder et al.*[6] | X-ray Crystal Structure, *Bau*[22] | X-ray Crystal Structure, *this work** | |
| Au-S, Å | 2.30 | | 2.277, 2.251 | 2.282, 2.268 |
| S-C, Å | | | 1.79, 1.78 | 1.818, 1.834 |
| S-Au-S, ° | ≅ 180 | 178.9, 169.4 | 178.8 | 176.03 |
| Au···Au, Å | 3.35, 5.8, 8.1 | 3.227, 3.485<br>5.78, 6.17 | 4.2 | 7.5 |
| Chemical Analyses * | Au:L, 1:1 | Au:L:Na:Cs, 2:2:2:1 | Au:L, 1:2 | Au:L, 1:2 |
| Observed Density * g $cm^{-3}$ | 2 component solid:<br>Upper: 2.731***RS***<br>Lower: 2.910***RS*** | 2.726, calculated[23] | 1.96 ***R*** | 2.05 ***R*** |

There is an intriguing parallel between the structures for $[AuL]_n$ and $AuL_2$ Self-organization into ***RR*** and ***SS*** double helices follows a balance of energy and entropy forces, and it would be expected for double helices in $[AuL]_n$, but not necessarily for the mononuclear complexes.. In $AuL_2$ the ***RR SS*** sorting also depends on the orientations and energetics across the Au(I) bond; the $AuL_2$ species might be viewed as $[AuL]_n$ in the limit of n = 1, and the self-organization apparently persists.

### Summary of gold and silver monomer and polymer structural data

Finally, it is interesting to compare what is now known of Au and Ag complexes of the two thiol ligands, thiomalic acid and **D**-penicillamine. Table VI is a brief overview of the types of structural data known for the complexes reviewed in this text. In addition, the gold complexes with mercaptoacetic acid and with 2-mercapto-5-methylbenzimidazole prepared by David Green are included For the complexes listed, crystal structures are now known for several mononuclear complexes and three of the polymers. Mass spectral data have been obtained for all the complexes, and tetramers (or in some cases higher polynuclear fragments) are observed for all the polymers, but not for any of the mononuclear complexes. Further work is continuing on the polymer $AuL_{II}$ and the monomers $Ag(L_I)_2$ and $Ag(L_{II})_2$.

### Conclusions

The data on related complexes support our findings for GST. We conclude that in addition to the mononuclear complex and the double helical polymer complex (crystalline solids), one of the principal components of GST in solution, and possibly in the solid, is a tetramer. To satisfy valence requirements, density measurements, and the chemical analyses, the tetranuclear species would have to be either a cyclic tetramer (supported also by the mass spectral data) with thiol ligands bridging pairs of gold atoms, or a tetrameric segment of an infinite chain. The formation of such tetranuclear species in aqueous solution is described by the equilibria shown for the general case and when n equals four:

$$n\,[Au(L_I)_2]^{5-} = n/2\,[L_I\,Au\,L_I\,Au\,L_I]^{7-} + n/2\ L_I^{\ 3-} = [(Au\,L_I)_n]^{2n-} + n\,L_I^{\ 3-}$$

or

$$4\,[Au(L_I)_2]^{5-} = 2\,[L_I\,Au\,L_I\,Au\,L_I]^{7-} + 2\,L_I^{\ 3-} = [(Au\,L_I)_4]^{8-} + 4\,L_I^{\ 3-}$$

Table VI: Structural Data of Au(I) and Ag(I) thiomalate and D-penicillamine Complexes [a]

| Polymer | Monomer | Structure | Mass Spectra |
|---|---|---|---|
| [Au(2-mercapto-5-methyl-benzimidazole)]$_n$ | | Crystal double helix* | Tetramers* |
| [Ag $L_I$]$_n$ | | | Tetramers[9] |
| [Ag $L_{II}$]$_n$ | | Crystal double helix* | Tetramers, pentamers hexamers* |
| | Au($L_I$)$_2$ | Crystal* | Monomers* |
| | Au($L_{II}$)$_2$ | Crystal* | Monomers* |
| [Au$L_I$]$_n$ | | WANES/WAX[6] Crystal, double helix[22] | Tetramers* |
| [Au(mercaptoacetic acid)]$_n$ | | | Pentamers, Hexamers* |

[a] Ligands: $L_I$ = thiomalate, $L_{II}$ = D-penicillamine;

* *this work*; [6] *Elder et al.*; [22] *Bau*

The GST solutions, and the solid (supported by density measurements), comprise more than one gold-containing species corresponding to different degrees of polymerization, the number and nature of these species depending on concentration, pH and nature of the cation. The variation in chemical analyses for Myochrysine™ samples reported earlier,[7] and the presence of free thiomalate in GST samples, are explained by equilibria of this type. Excess ligand also accounts for the yellow component formed on heat sterilization of the drug: the ligand oxidizes readily to form a disulfide (or a more highly oxidized form). Structural data now support the existence of three gold thiomalate complexes as main components of GST (and of Myochrysine™, which contains additional chemical components)--a mononuclear complex, a tetranuclear complex, and a polymeric complex. During the drug synthesis, as the product in solution is being spun in the rotary evaporator, there will be a distribution of oligomer and polymer sizes. As the solution becomes solid, the monomer or mononuclear species crystallizes first. The polymer, much more difficult to crystallize, is double helical and is very similar to the silver D-penicillamine polymer non-molecular crystal. The repeat unit in the GST helical polymer is tetranuclear, and it apparently forms the tetranuclear complex observed by ESI-MS. According to the biological tests, the polymer is one of the active components of the drug Myochrysine™. The tetranuclear and mononuclear complexes have not been tested; if they are pharmacologically effective, they have the advantage of being easier to prepare in optically pure form as well as being easier to purify chemically by crystallization. The adverse side effects have more than one source; namely, the optically impure gold(I) thiomalate complexes and the oxidized forms of the ligand thiomalate.

**Acknowledgements**

I wish to acknowledge, with thanks, the contributions of several people: My fellow co-directors in the Laboratories for Inorganic Medicine, (L.I.M.), are Drs. C. J. L. Lock, (the late, L.I.M. founder), R. A. Bell, W. W. Buchanan, and W. F. Kean. Dr. Alfred Bader supplied chemicals at cost and advice enabling us to develop a single enantiomer synthesis for thiomalic acid. Rhone Poulenc Pharma and May and Baker provided samples of Myochrysine™. Dr. C. F. Shaw III, Dr. R. C. Elder, and Dr. R. V. Parish have been international collaborators for many years. Mary Duarte, laboratory manager, Daren LeBlanc and David Green, graduate students and Zhixian Wang, Postdoctoral Fellow, carried out the synthetic and structural work on gold complexes described here. Drs. R. W. Smith and J. F. Britten assisted with mass spectra and with X-ray data. Dr. Kerry Beal and Ms. Janice Ritshke carried out biological tests. The Arthritis Society of Canada, the National Sciences and Engineering Research Council of Canada, the Medical Research Council of Canada, and the McMaster University Hospital funded the work. H. E. H.-L. was R. Samuel McLaughlin Career Scientist in Gerontological Research during part of the course of this research.

**References**

1 Pathaneni, S.S.; Desiraju, G.R. *J. Chem. Soc., Dalton Trans.* **1993**, 319-322.
2 Pyyckko, P. *Chem. Rev.* **88**, 563-594 (1988).
3 Schmidbaur, H.; Dziwok, K.; Grohmann, A.; Müller, G. *Chem. Ber.* **122**, 893-895 (1989).
4 LeBlanc, D.J. *Ph.D. Thesis, McMaster University,* (1996).
5 Lock, C.J.L. *Inflammopharmacology,* **4**, 1-16 (1996).
6 Elder, R.C.; Eidsness, M.K. *Chem. Rev.*, **87**, 1027-1046 (1987).
7 Harvey, D.A.; Howard-Lock, H.E.; Lock, C.J.L. *Canadian Chemical News*, **40***(10)*, 19-21 (1988).
8 Duarte, M.; Howard-Lock, H.E.; Lock, C.J.L. *Unpublished results*, (1984-1988).
9 Nomiya, K.; Kondoh, Y.; Nagano, H.; Oda, M. *J. Chem. Soc., Chem. Commun.* **1995**, 1679-

1680.
[10] LeBlanc, D.J.; Smith, R.W.; Wang, Z.; Howard-Lock, H.E.; Lock, C.J.L, (the late). *J. Chem Soc., Dalton Trans.*, **1997**, 3263-3267.
[11] LeBlanc, D.J.; Britten, J.F.; Wang, Z.; Howard-Lock, H.E.; Lock, C.J.L, (the late). *Acta Cryst.* **C53**, 1763-1765 (1997).
[12] sab, A.A.; Sadler, P.J. *J. Chem. Soc., Dalton Trans.* **1981**, 1657-1662.
[13] Schröter, I; Stränle, J. *Chem Ber.*, **124**, 2161-2164 (1991).
[14] LeBlanc, D.J.; Lock, C.J.L. (the late). *Acta Cryst.* **C53**, 1765-1768 (1997).
[15] Howard-Lock, H.E.; Lock, C.J.L.; LeBlanc, D.J. *Canadian Society for Chemistry Conference, Winnipeg, ON, Canada* (1994).
[16] Howard-Lock, H.E.; LeBlanc, D.J.; Lock, C.J.L, Smith, R.W.; Wang, Z.; *J. Chem. Soc., Chem. Commun.* **1996**, 1391-1392.
[17] Bonasia, P.J.; Grindelberger, D.E.; Arnold, J. *Inorg. Chem.* **32**, 5126-5131 (1993).
[18]. Wojnowski, W.; Becker, B.; Sassmannshausen, J.; Peters, E.M.; Peters, K.; von Schnering, H.G. *Z. Anorg. Allg. Chem.* **620**, 1417-1421 (1994).
[19] Crespo, O.; Gimeno, M.C.; Jones, P.G.; Laguna, A. *J. Chem. Soc., Chem. Commun.* **1993**, 1696-1697.
[20] LeBlanc, D.J. *Ph.D. Thesis, McMaster University*, 131-133 (1996).
[21] Bell, R.A. *Personal Communication* (1998).
[22] Bau, R. *J. Am. Chem. Soc.* **120**, 9380-9381 (1998).
[23] Brown, I.D. *Personal Communication* (Mar. 17, 1999).

**Received: October 8, 1998 - Accepted in final form: April 26, 1999**

